# The interplay of influencing factors in physical activity promotion for older men: insights through the capability approach

**DOI:** 10.1186/s12889-026-29091-5

**Published:** 2026-08-22

**Authors:** Tizian Schuck, Julika Loss, Susanne Tittlbach

**Affiliations:** 1 BaySpo – Bayreuth Center of Sport Science, Chair of Social and Health Sciences of Sport, University of Bayreuth, Universitätsstraße 30, Bayreuth, 95447 Germany; 2 DSS – Department of Sport Science and Sport, Chair of Education in Sport, Friedrich-Alexander-University of Erlangen-Nürnberg, Gebbertstraße 123b, Erlangen, 91058 Germany; 3 Department Epidemiology and Health Monitoring, Robert Koch Institute (RKI), Berlin, Germany

**Keywords:** Capabilities, Active lifestyle, Community, Conversion factors, Structural Equations Models (SEM)

## Abstract

**Background:**

The capability approach has gained increasing attention in the context of health and physical activity promotion due to its focus on individuals’ freedoms to achieve desired outcomes. Its empirical application remains limited, particularly among older men, a group often underrepresented in physical activity promotion. This study applies the capability approach as a theoretical framework to investigate the relationship between individual and socio-structural conversion factors, perceived capabilities for an active lifestyle, and actual physical activity behavior among men aged 50 and older in a community setting.

**Methods:**

Based on a cross-sectional survey (*N* = 316), partial least squares structural equation modeling was used to assess associations between individual and socio-structural conversion factors (e.g., individual barriers, self-efficacy, structural barriers, and social support from family and friends, as well as a supportive neighborhood environment) and capabilities for an active lifestyle, and between capabilities and actual physical activity behavior. Explanatory power and predictive power were evaluated, and alternative model specifications compared.

**Results:**

Individual (*r* = -.38, f^2^ = .17) and structural (*r* = -.26, f^2^ = .07) factors are significantly associated with perceived capabilities for an active lifestyle (*R*^2^ = .28). Higher capabilities for an active lifestyle were positively related to higher actual physical activity levels (*r* = .32, f^2^ = .12), although explanatory power for actual physical activity behavior was modest (*R*^2^ = .10). The estimated model demonstrated good out-of-sample predictive power and superior fit compared with alternatives.

**Conclusion:**

This study supports the application of the capability approach in physical activity promotion, demonstrating that individual and structural factors interact to shape older men’s perceived capabilities for an active lifestyle. Social factors were not retained in the final model. The findings indicate that physical activity promotion should be tailored to specific needs of the target group. The findings emphasize that physical activity promotion should not only aim to increase behavioral outcomes, but also to ensure equal capabilities for all individuals, while acknowledging the role of conversion factors. Fostering such equitable capabilities for an active lifestyle, particularly for underrepresented populations in physical activity promotion, can be considered as a success in itself. Future interventions should account for diverse conversion factors and group-specific needs.

## Introduction

With the adoption of the Ottawa Charter, the World Health Organization [1] established the goal of achieving health for all on a global scale. This ambitious aim emphasizes health equity by reducing disparities and ensuring equal opportunities for health. The Charter underscores the importance of health and physical activity promotion as a process centered on self-empowerment and supportive environments, particularly within communities. These health targets remain highly relevant today, as reflected in Sustainable Development Goal 3: “Ensure healthy lives and promote well-being for all at all ages” [2, 3], which emphasizes reducing health inequalities and addressing environmental factors of health.

Despite ongoing international efforts, older men are identified as a particularly underrepresented group in health and physical activity promotion in Germany [4]. This is due to several factors like unfavorable health risk profiles [5], higher levels of social isolation [6], and low participation in health-enhancing physical activity interventions [5, 7]. While men are generally more active than women in youth and early adulthood, their physical activity levels decline sharply, leading to convergence in middle and older age [8]. Furthermore, social inequalities continue to shape physical activity behavior, with lower educational levels negatively associated with physical activity across all age groups [8].

**Fig. 1:**
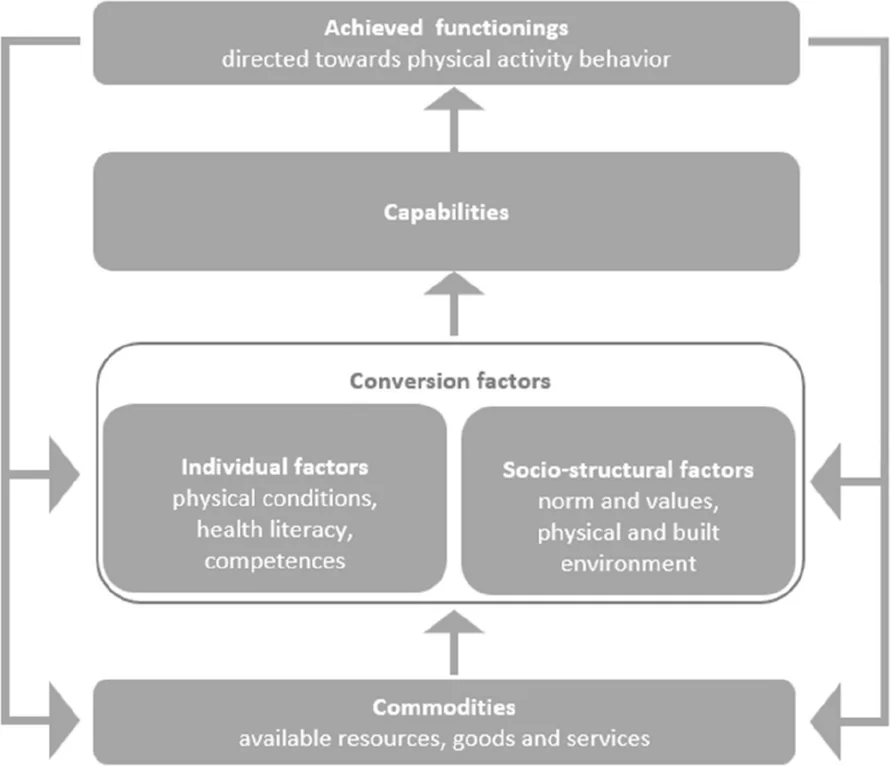
Model of the capability approach (adopted by Robeyns [11] and Frahsa et al. [14])

The capability approach – developed by Sen [9] and Nussbaum [10], and further elaborated by Robeyns [11] – offers a normative framework for evaluating well-being and social justice beyond economic indicators. The approach focuses on people´s freedom and their capabilities to choose and pursue the lives they value. Applied to health and physical activity promotion, the capability approach provides a person-centered perspective that emphasizes empowerment and the conditions enabling individuals to pursue active lifestyles [12, 13], which is consistent with the principles of the Ottawa Charter [1]. Rather than focusing solely on observable behavior, the approach highlights perceived capabilities for leading an active life. These capabilities refer to individuals’ perceived opportunities and possibilities to engage in physical activity and are shaped not just by individual or socio-structural factors on their own, but by how individuals perceive and experience the complex interplay between these factors (Fig. 1).

Within the capability approach, different interpretations and operationalizations have been developed depending on the specific research context [15]. In the field of health promotion and physical activity, the capability approach has been adapted to understand how available means, individual characteristics, and contextual conditions interact to shape individuals’ opportunities to engage in health-related behaviors [14, 16]. In the present study, we follow the operationalization applied within the Capital4Health research context [17], in which individual factors such as health literacy, as well as social and environmental conditions, are conceptualized as conversion factors influencing how available means are transformed into capabilities.

Within this framework, commodities refer to the means available to individuals, including material and immaterial goods as well as available services [14]. In the context of physical activity, commodities may include available resources and opportunities that provide general conditions for an active lifestyle. However, the availability of such means alone does not determine individuals’ opportunities for action. Rather, capabilities emerge through a conversion process in which individual, social, and environmental conversion factors shape how available means are accessed, experienced, and transformed into meaningful possibilities for action [11]. For example, similar environmental resources may result in different capability sets depending on individuals’ personal characteristics, social circumstances, or contextual conditions. Consequently, different combinations of conversion factors may lead to differences in perceived capabilities for leading an active lifestyle. From a health promotion perspective, the capability approach therefore emphasizes the importance of creating conditions that enable all individuals to develop comparable opportunities and freedoms to engage in physical activity, rather than focusing solely on changing individual behavior.

While individual and socio-structural correlates of physical activity have been widely studied [18, 19], their role in shaping perceived capabilities within the framework of the capability approach has received little empirical attention, particularly among underrepresented subgroups such as older men. Initial conceptual groundwork was laid by Sauter et al. [20], who used exploratory qualitative research to identify relevant individual and socio-structural capabilities for an active lifestyle among older adults. Building on this, Boyer et al. [21] recently developed and validated a questionnaire to assess these capabilities. However, these contributions do not examine which factors are more crucial in shaping capabilities. The present study addresses this empirical gap by applying the capability approach to investigate which type of conversion factors – individual or socio-structural – plays a more crucial role in shaping perceived capabilities for an active lifestyle among older men.

### The capability approach in health and physical activity promotion

In the context of health and physical activity promotion, the capability approach complements other theoretical frameworks like the behavior change wheel [22] or ecological models [23] by adding a justice-oriented perspective [13]. Unlike models that primarily frame behavior change as the result of reducing individual barriers or increasing environmental attractiveness, the capability approach questions this linear logic. For instance, Till et al. [24] argue that conventional approaches often focus too narrowly on outcomes while neglecting the enabling or constraining social or environmental conditions [25]. Instead, the capability approach shifts the focus to fostering individual capabilities, expanding people’s freedom to choose an active lifestyle.

In Germany, the practical relevance of the capability approach has been demonstrated in projects such as the Capital4Health research consortium, which employed the capability approach as an umbrella framework across different settings and populations. All sub-projects confirmed the approach’s utility as a bridging framework for interdisciplinary collaboration [14]. Similarly, the BIG project aimed to develop low-threshold physical activity programs for socioeconomically disadvantaged women. A systematic categorization of project components along capability dimensions revealed underlying pathways of change [16]. Despite these promising examples, these applications of the capability approach remained largely conceptual.

A central strength of the capability approach lies in its recognition of conversion factors, which enable or constrain capabilities. These include individual factors such as motivation and self-efficacy, as well as socio-structural factors like social support, a supportive neighborhood environment or access to infrastructure. Importantly, the capability approach does not prioritize one type of factor over the other, but instead stresses their interaction [26]. In practice, however, interventions often require prioritization [27]. For example, structural strategies like enhancing community readiness [28] or building organizational capacity [29] primarily target external conversion factors. In contrast, individual-focused strategies aim to strengthen internal factors by addressing motivational barriers or improving health competencies [30]. Although both strategies aim to promote active living, prioritizing one factor over the other is often not empirically justified.

While the integration of internal (individual) and external (socio-structural) factors is widely acknowledged as best practice [27, 31], there remains a notable lack of quantitative research that empirically examines their relative influence on perceived capabilities, particularly for underrepresented groups such as older men. Addressing this gap can help inform more targeted and equitable health and physical activity promotion strategies.

### Hypotheses and model development

To develop a theoretical model, existing health and physical activity research findings must be interpreted through the lens of the capability approach. Garcia et al. [19] provide a comprehensive overview of physical activity determinants, distinguishing between individual (e.g., motivation), social (e.g., social support), and structural (e.g., perceived walkability) factors. Similarly, Carlson et al. [32] and Wang et al. [33] highlight the interconnectedness of these conversion factors in shaping physical activity behavior. For community-dwelling older adults in particular, the built environment (e.g., walkable neighborhoods) has been consistently linked to higher levels of leisure-time physical activity [34, 35].

Based on this empirical evidence and the conceptual foundation of the capability approach, the following hypotheses are proposed:

- H1: Individual and socio-structural factors, such as higher self-efficacy and fewer barriers, are positively associated with greater capabilities for an active lifestyle when considered simultaneously.
- H2: Individual and socio-structural factors differ in the strength of their association with capabilities for an active lifestyle.
- H3: Capabilities for an active lifestyle are positively related to actual physical activity behavior.

## Methods

### Design

This study is a secondary analysis of the Action for Men (A4M) project data (funding code BMBF 01EL1421D; [36]), which was embedded in the CAPITAL4HEALTH research network [17]. The study used cross-sectional survey data collected in 2016 from community-dwelling men aged 50 years and older living in the rural municipality of Sulzbach-Rosenberg (Upper Palatinate region in Bavaria, Germany). The A4M study was reviewed and approved by the research ethics committee at the University of Bayreuth (Approval No.: O 1305/1-GB). Written informed consent was obtained from all participants before participating in the study. Participation was voluntary, and withdrawal was possible at any time.

### Characteristics of participants

A total of 316 men (age: M = 64.87, SD = 9.70) completed the survey. Of these, 130 are in the low education group (41.1%), 77 in the medium education group (24.4%), and 109 in the high education group (34.5%). 44 men live alone (14.0%), and 272 men live in a family household or with other people (86.1%). Sociodemographic characteristics of the study sample are presented in Table 1.

**Table 1:** Sociodemographic characteristics of the study sample

Characteristic		Total sample (*N* = 316)
Age, years (*Mean* ± *SD*)		64.87 ± 9.70
Age groups, *n* (%)	50–59 years	113 (35.8%)
	60–69 years	99 (31.3%)
	70–79 years	79 (25.0%)
	≥ 80 years	25 (7.9%)
Educational level, *n* (%)	Low education	130 (41.1%)
	Medium education	77 (24.4%)
	High education	109 (34.5%)
Living arrangement, *n* (%)	Living with family/other people	272 (86.1%)
	Living alone	44 (13.9%)

### Operationalization of constructs

The survey consists of self-assessments and validated measures that were selected to operationalize key elements of the capability approach, particularly perceived capabilities and relevant individual, social, and structural conversion factors. Sociodemographic characteristics such as age, educational level, and living arrangement are included and reflect the basic commodities within the capability approach.

Individual conversion factors are assessed through items measuring self-efficacy and individual barriers [37, 38]. Self-efficacy was reverse-coded, so that higher values in the individual conversion factors mean fewer self-efficacy and higher barriers (e.g., more laziness or lower interest in physical activity). Socio-structural conversion factors capture barriers related to the physical and social environment, including limited access to physical activity opportunities, neighborhood characteristics, transportation infrastructure, and insufficient social support [39]. Capabilities for an active lifestyle are measured using three items that capture individuals’ subjective perception of their opportunities to live a healthy life, engage in leisure-time physical activity, and organize their leisure time according to their own needs and preferences [40–42]. For example, participants responded to statements such as, “I perceive the opportunity in my life to be physically active in my leisure time as …”, thereby explicitly reflecting their personal experience and perception of their capabilities for action. Additionally, actual physical activity behavior (achieved functioning) is assessed by examining the frequency and intensity of leisure-time physical activity and active travel [43]. All items used demonstrate acceptable Cronbach’s alpha values (see Table 5 in the Appendix).

### Type of statistical analysis

Statistical analyses were conducted using R Version 4.4.3 (R Foundation, Vienna, Austria). Data preparation and partial least squares structural equation modeling (PLS-SEM) analyses were performed using the seminr package, while missing data were handled using the mice package. Prior to analysis, missing data were assessed. The proportion of missing values was low (2.84% of all possible observations), and missingness was assumed to be missing at random. Missing values were handled using multiple imputations by chained equations (MICE) with predictive mean matching (PMM).

For inferential analyses, PLS-SEM was applied, which is well suited for prediction-oriented research within the capability approach [44]. Statistical significance was evaluated using nonparametric bootstrapping with 1,000 resamples. A significance level of α = 0.05 was applied, with bootstrap t-values exceeding 1.96 indicating statistically significant relationships. Model evaluation included indicator loadings, construct reliability, convergent validity, discriminant validity (Fornell–Larcker criterion and HTMT), standardized coefficients, effect sizes (f^2^), and explained variance (R^2^). Out-of-sample predictive performance was assessed using the PLSpredict procedure. A disjoint sample prediction approach with 10-fold cross-validation was applied to evaluate the model’s predictive ability [45]. Prediction accuracy was evaluated using the root mean squared error (RMSE) and mean absolute error (MAE) and compared with the benchmark ordinary least squares (OLS) regression model implemented in the PLSpredict procedure. Additional model specifications were compared using the Bayesian Information Criterion (BIC) and BIC-based Akaike weights. The primary capability-based model was compared with alternative model specifications excluding capabilities or treating capabilities as parallel predictors rather than as a mediating construct [46, 47].

## Results

This section presents the results of PLS-SEM, which are based on the theoretical framework of the capability approach and are consistent with the proposed hypotheses. The findings are organized into three analytical levels: the measurement model (outer model) assesses the quality of constructs and indicators, the structural model (inner model) addresses hypothesis testing, and the overall model evaluates predictive accuracy and model fit.

### Measurement model (outer model)

The measurement model serves to evaluate the reliability and validity of the latent constructs and their associated indicators. As this study employs a reflective measurement approach, the assessment follows established reliability and validity criteria [44]. The sociodemographic variables had no significant relationship with conversion factors or other model dimensions and are therefore not included in the following analyses.

At the item level, indicator reliability was examined through factor loadings. Loadings above 0.70 are generally considered acceptable, while loadings between 0.40 and 0.70 should be interpreted with additional reliability and validity criteria [48]. Communalities, corresponding to squared factor loadings, reflect the proportion of variance explained by each indicator and should exceed 0.50 [44]. During the model development process, several items were excluded due to insufficient reliability or limited relevance to the target group. These included individual barriers such as inactivity due to health restrictions or lack of time, structural factors like safety on footpaths and all social support indicators (e.g. lack of family support, lack of support from neighbors or friends, and no supportive neighborhood environment). In the final model, only individual and structural factors were retained. Within the physical activity behavior construct, two items showed factor loadings slightly below the 0.708 threshold; however, since they exceeded the minimum cut-off of 0.40 and satisfied all other quality criteria, they were included in the final analysis [48]. An overview of the final items included in the model is provided in Table 2.

**Table 2:** Outer model results

Construct	Item	Unit/Scale	M	SD	FL	*h*^*2*^	VIF	*α*	CR	AVE
Individual factors	Maintenance self-efficacy	1—6	2.66	1.55	.85	.73	2.37	.82	.88	.58
	Motivational self-efficacy	1—6	3.21	1.63	.74	.55	1.87			
	No interest	1—7	2.12	1.44	.76	.58	1.69			
	Uncertain about physical abilities	1—7	1.96	1.23	.75	.57	1.94			
	Laziness	1—7	2.28	1.49	.70	.49	1.62			
Structural factors	Lack of sports facilities	1—7	2.10	1.34	.76	.58	1.79	.81	.87	.57
	Lack of public transportation	1—7	2.13	1.28	.78	.61	1.62			
	Demands of the neighborhood	1—7	1.75	.94	.72	.51	1.53			
	Design of the neighborhood	1—7	2.43	1.49	.76	.58	1.66			
	Inconvenient schedule	1—7	2.43	1.48	.75	.57	1.60			
Capabilities	Capabilities for activity	1—7	5.65	1.07	.92	.85	2.38	.85	.91	.77
	Capabilities for health	1—7	5.83	.96	.86	.75	2.06			
	Capabilities for leisure time	1—7	5.59	1.15	.85	.72	2.00			
Actual physical activity behavior	Leisure time (intensive)	Min/week	242.55	162.91	.82	.67	1.12	.51	.74	.50
	Leisure time (moderate)	Min/week	207.29	189.18	.68	.47	1.15			
	Active travel	Min/week	248.24	248.93	.60	.35	1.09			

*AVE* Average variance extracted, *α* Cronbach alpha, *CR* Composite Reliability, *FL* Factor loading, *h*^*2*^ Communalities, *M* Mean, *SD* Standard deviation, *VIF* Variance Inflation Factor

Collinearity was assessed via the variance inflation factor (VIF), despite this being more common in formative models. All VIF values fell within the recommended range of 0.20 to 3.00, suggesting no multicollinearity issues [44]. Internal consistency reliability was evaluated using both Cronbach’s alpha [49] and composite reliability [50]. While Cronbach’s alpha remains widely used, it assumes equal indicator loadings, a limitation addressed by the more flexible composite reliability [44, 51]. All constructs — individual factors, structural factors, and capabilities — exceeded the 0.70 threshold for both measures. For physical activity behavior, reliability was only acceptable based on composite reliability.

Convergent validity was confirmed through average variance extracted (AVE), with all constructs meeting the threshold value of 0.50 [48]. Discriminant validity was assessed using the Fornell-Larcker criterion [44, 52] and the Heterotrait-Monotrait (HTMT) ratio [53]. For all constructs, the square root of the AVE exceeded inter-construct correlations, and all HTMT values were below the conservative threshold of 0.85 (Tables 6 and 7 in the Appendix). Both criteria support adequate discriminant validity of the model.

### Structural model (inner model)

The structural model involves hypothesis testing based on the standardized coefficients between the latent constructs (Table 3). As a preliminary step, multicollinearity was assessed at the construct level using VIF. None of the VIF values exceeded the recommended threshold of 3.0, indicating no multicollinearity concerns [44].

**Table 3:** Inner model results

Hypothesis/Paths		β	Bootstrap SD	t-value	2.5% CI	97.5% CI	p	f	Supported
H1:	IF → CA	-.38	.05	−7.45	-.47	-.28	**	.17	Yes
H2:	SF → CA	-.26	.06	−4.50	-.38	-.15	**	.07	Yes
H3:	CA → PAB	.33	.04	7.51	.24	.41	**	.12	Yes

*CA* Capabilities, *CI* Confidence interval, *IF* Individual factors, *PAB* physical activity behavior, *SD* Standard deviation, *SF* Structural factors, *β* standardized coefficient, *f*^*2*^ effect size

P significance level, ** *p* <.01

To determine the significance and relevance of the path relationships, bootstrapping procedures were employed to generate standard errors and calculate corresponding t-values [54]. The analysis revealed that both individual and structural factors are significantly associated with capabilities for an active lifestyle. Specifically, higher individual barriers and fewer self-efficacy, along with higher structural barriers, were associated with fewer perceived capabilities. Furthermore, capabilities for an active lifestyle are significantly related to physical activity behavior. This means, if individuals perceive more freedom and capabilities to actively shape their own leisure time, they are more willing to be physically active.

**Fig. 2:**
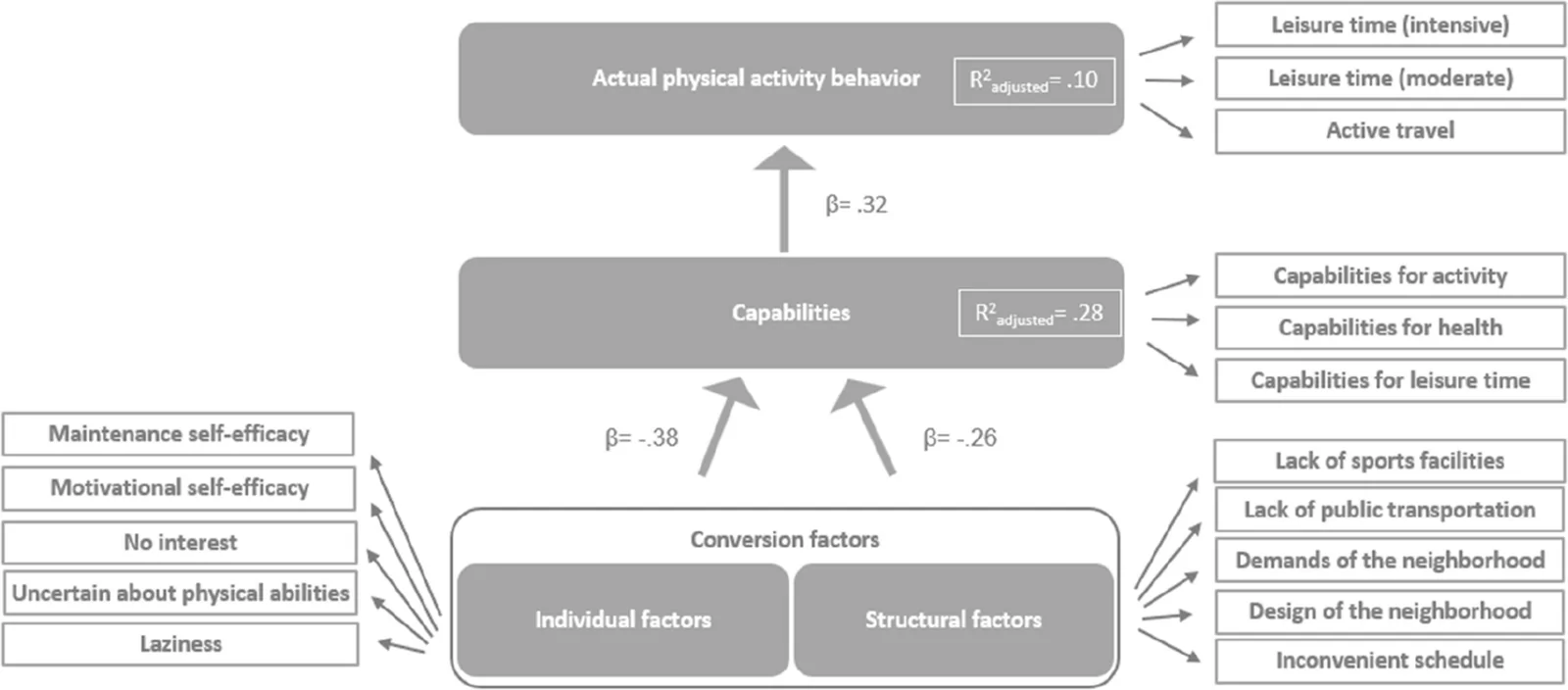
Estimated structural model of the capability approach

The model’s explanatory power was assessed using the coefficient of determination (R^2^). Conversion factors accounted for 28% of the variance in capabilities, whereas capabilities explained 10% of the variance in physical activity behavior, respectively functioning (Fig. 2). These *R*^2^ values should be interpreted cautiously, as the value depends on the number of predictor variables [44]. To complement the explanatory power, effect sizes were calculated to rank the relative association of each predictor. As shown in Table 3, individual factors demonstrated a medium association, while structural factors had a small association on capabilities [44].

### Overall model

The overall model’s robustness was assessed with the out-of-sample predictive power and comparative model evaluations.

For the out-of-sample predictive power, the PLS model was compared against a linear regression model (Table 4). The PLS model achieved lower RMSE and MAE values for all physical activity behavior indicators, indicating slightly superior out-of-sample predictive performance compared with the linear benchmark model [44].

**Table 4:** Summary of the model´s predictive power

Items	Unit	RMSE		MAE	
		**PLS out-of-sample**	**LM out-of-sample**	**PLS out-of-sample**	**LM out-of-sample**
Leisure time (intensive)	Min/week	156.60	158.35	112.51	112.84
Leisure time (moderate)	Min/week	186.69	191.36	117.47	122.24
Active travel	Min/week	246.20	251.30	147.56	150.88

*LM* Linear model, *MAE* Mean absolute error, *PLS* Partial least squares, *RMSE* Root mean squared error

**Fig. 3:**
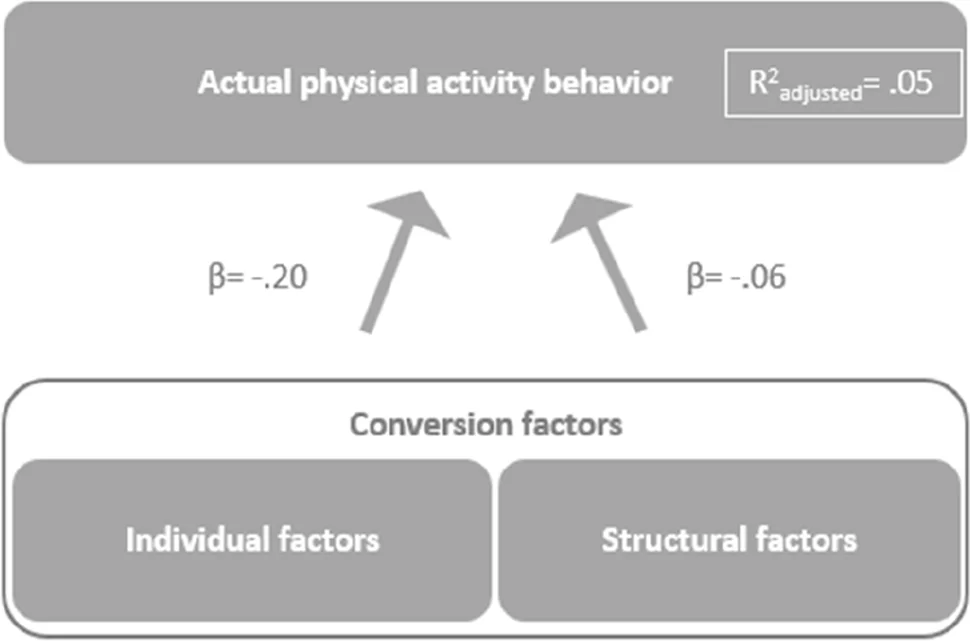
Estimated structural model based on a model without capabilities

In addition to predictive power, model comparisons were conducted to evaluate the quality of different model configurations emerging from different theories. The primary model based on the capability approach (Fig. 2), was compared with a model without capabilities (Fig. 3) in which capabilities were omitted. This more traditional model assumes direct associations of individual and structural factors on physical activity behavior. While this more traditional model showed a slightly lower explained variance in predicting physical activity behavior, the path from structural factors to physical activity behavior was no longer significant, with only individual factors remaining relevant.

**Fig. 4:**
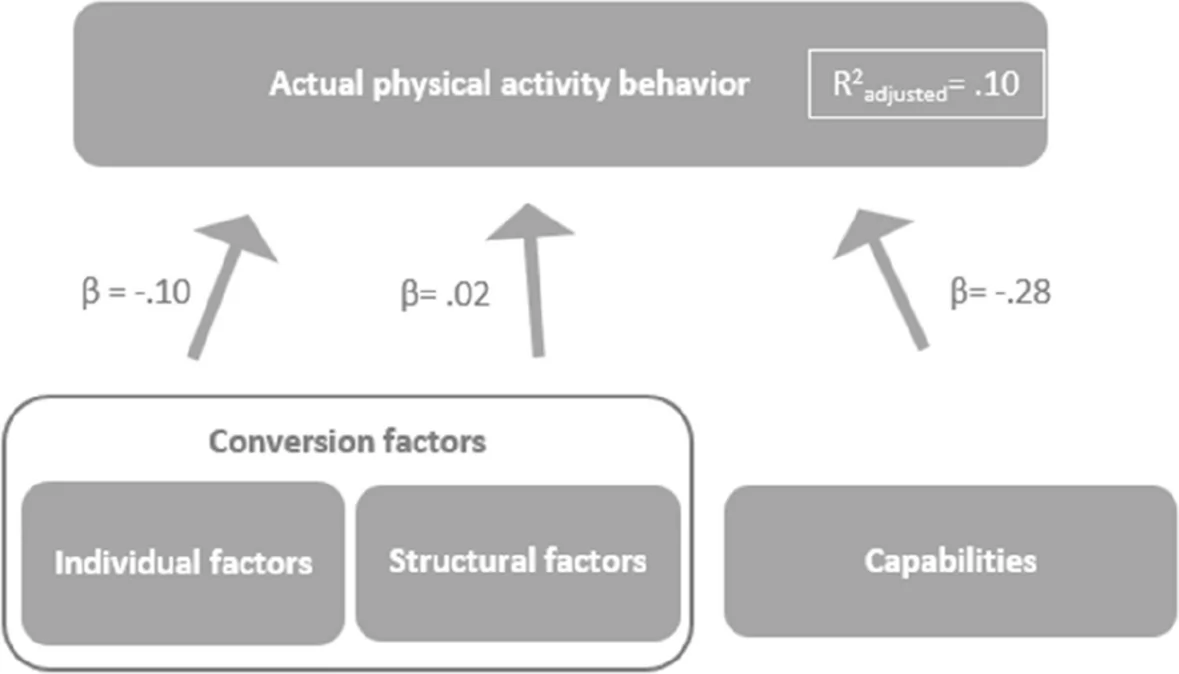
Estimated structural model based on a model without hierarchies

A third model without hierarchies (Fig. 4) was included in the model comparison process, in which capabilities were positioned alongside conversion factors rather than as a mediating construct. The explained variance in predicting physical activity behavior remains consistent with that of the primary model. However, in this model, capabilities and individual factors are significantly related to physical activity behavior, while structural factors show no association.

All three models explained a comparable share of variance in physical activity behavior. However, when evaluating the model, the primary capability-based model (BIC weights = −23.86) and the model without hierarchies (BIC weights = −14.62) achieved lower values in comparison to the model without capabilities (BIC weights =—0.61), indicating better model fits. When considering relative model likelihoods, the primary capability model emerged as the most plausible data-generating model and would be preferred in nearly all scenarios (BIC based Akaike weights = 99.02%).

## Discussion

The present study examined how individual and socio-structural conversion factors are associated with perceived capabilities for an active lifestyle and actual physical activity behavior among men aged 50 and older, using the capability approach as a theoretical framework. Higher individual barriers and lower self-efficacy, along higher structural barriers, were linked to fewer perceived capabilities, which in turn were positively associated with activity levels. Individual factors had a stronger association than structural factors, while social factors were not retained in the final model. Although capabilities explained more variance in actual physical activity behavior than direct conversion factors, the explained variance in actual physical activity behavior was modest. Model comparisons confirmed that the capability-based framework provides a better fit than models without hierarchical mediation or without capabilities.

### Advancing health and physical activity promotion

The findings largely support the proposed hypotheses. Consistent with H1, both individual and structural factors exert a positive association on capabilities related to an active lifestyle. In contrast, social factors did not make it into the final model, suggesting their role may be less pronounced within this context. Regarding H2, the results indicate a difference in the relative strength of the relationships, highlighting that individual factors are more related to shaping capabilities than structural factors. In line with H3, enhanced capabilities are positively associated with actual physical activity behavior.

These results emphasize the practical relevance of the capability approach in the context of physical activity promotion. Although the capability approach has been widely applied in well-being research, its potential in health and physical activity promotion is still emerging [15]. Notable exceptions such as Lewis [55, 56], Ferrer and Carrasco [57], and Ferrer et al. [58] demonstrate the value of capturing both individual and structural factors in physical activity promotion. These studies underscore the role of conversion factors in enabling individuals to transform individual and structural factors into functioning.

The primary model based on the capability approach (Fig. 2), which demonstrated the best model fit and strongest statistical power, highlights the interplay between individual and structural factors in shaping perceived capabilities. In contrast, the model without capabilities (Fig. 3) indicated that only individual factors, such as higher individual barriers and lower self-efficacy, are significantly related to physical activity behavior. However, this narrower perspective contradicts widely accepted recommendations that emphasize the integration of individual and socio-structural factors [19, 31, 33]. Accordingly, this model yielded lower explanatory power and reduced statistical significance.

A similar pattern was observed in the model without hierarchies, where capabilities for an active lifestyle were placed at the same hierarchical level as conversion factors (Fig. 4). This non-hierarchical structure also failed to enhance explanatory power and again resulted in weaker statistical performance compared to the primary model.

Taken together, the findings reinforce two key insights: First, they empirically support the hierarchical logic of the capability approach, where perceived capabilities act as a bridge between conversion factors and physical activity behavior. Second, they emphasize the importance of maintaining a comprehensive perspective that acknowledges the complex interplay among various conversion factors [24]. While individual factors like self-efficacy remain essential, they must be contextualized within broader structural environments [19, 32].

### Beyond behavior change: The freedom of choice

The complexity of promoting physical activity is evident in this study. The link between capabilities and actual physical activity behavior is less pronounced than the connection between conversion factors and capabilities. This raises the question of whether success in physical activity promotion should be evaluated by an increase in physical activity or by ensuring equal capabilities for participation [26].

A central contribution of the capability approach is its focus on fostering equal capabilities rather than simply achieving behavioral outcomes. The decision to engage in physical activity remains personal, varying from case to case, and always encompasses a range of behaviors, including both health-promoting and risk-related choices [13]. From a capability perspective, expanding opportunities for physical activity represents a valuable outcome in itself, independent of whether individuals ultimately choose to be physically active [16]. Individuals may value having the freedom and opportunity to engage in physical activity without necessarily selecting this option. Consequently, physical activity promotion should not aim solely to increase physical activity levels but to create conditions that enable individuals to make self-determined choices regarding physical activity [14, 59]. The findings reinforce the idea that capabilities do not necessarily have to convert into functioning, and that access to conversion factors alone does not induce behavior change [11, 14]. Nevertheless, strengthening conversion factors can expand individuals’ capabilities and thereby enhance their freedom to choose whether and how to engage in physical activity.

This distinction is particularly relevant for underrepresented groups in health and physical activity promotion. A best-practice example is provided by Till et al. [16] through the BIG project involving socially disadvantaged women. Their implemented interventions showed that improvements in individual and socio-structural factors can increase perceived capabilities for an active lifestyle and lead to higher levels of physical activity. Although the present study focuses on older men in the A4M project, who represent another underrepresented subgroup, similar mechanisms may apply. This suggests that tailored interventions targeting conversion factors could be effective across diverse populations. Additionally, Till et al.’s [16] investigation of collective-level effects highlights an important area not covered in the present study but worth exploring in future research. Such collective and social processes may contribute to individuals’ agency by shaping their ability to act upon available opportunities for physical activity, particularly among men in community settings [12].

### Rethinking the relationship of commodities and conversion factors

Contrary to common assumptions and previous studies [8, 60], sociodemographic variables (age, educational level, living arrangement) showed no meaningful association with conversion factors, capabilities, or physical activity behavior in the present study. This aligns with the conceptual logic of the capability approach, where sociodemographic variables can be interpreted as commodities – contextual living conditions that potentially shape conversion factors but do not directly determine capabilities or physical activity behavior [11]. For men aged 50 and older in community settings, the perception of capabilities to engage in physical activity, particularly in leisure and active travel contexts, appears to be shaped more strongly by individual and structural factors. Importantly, these conversion factors seem largely independent of social position [12].

While social inequalities — such as lower education and residence in underserved areas — are commonly linked to lower levels of physical activity due to reduced access to enabling environments [61], this association was less pronounced in the present sample. Alternatively, such inequalities might already be captured through the structural factors, reducing the additional explanatory value of sociodemographic variables.

### Practical implications and future directions

The findings of this study suggest that physical activity promotion among older men should not solely focus on increasing activity levels but also on strengthening the conditions that enable individuals to develop capabilities for an active lifestyle. While capabilities do not necessarily translate into physical activity behavior, creating equitable opportunities for participation represents a valuable goal in itself.

From a practical perspective, interventions based on the capability approach should address both individual and structural factors. At the individual level, practitioners should identify perceived barriers and facilitators and strengthen relevant individual factors through tailored support that enhances self-efficacy for an active lifestyle. At the structural level, efforts should focus on creating accessible and inclusive environments while ensuring that these opportunities are actually perceived as meaningful by the target group. Participatory approaches, such as involving community stakeholders and members of the target group in identifying local barriers, mobilizing existing resources, and co-developing context-specific physical activity programs, may represent a promising strategy for capability-building [36, 62].

However, the relevance of specific conversion factors may depend on the target population and context. Mitchell et al. [15] pointed out that the capability approach offers a robust conceptual framework that is flexible enough to be adapted to specific contexts. Similarly, Ferrer et al. [58] emphasized that individuals require different forms of support to create equitable capabilities for engaging in physical activity. The exclusion of social factors, such as family or community support, in this study suggests a need to adapt the capability approach to the target group and raises the question of whether all conversion factors are universally relevant across different population groups.

For older adults in particular, Sauter et al. [20] and Boyer et al. [21] identified relevant individuals and socio-structural capabilities for an active lifestyle and introduced new approaches to operationalize components of the capability approach. Future research should further investigate how different conversion factors, capabilities, and agency-related processes interact across diverse population groups and community settings.

## Strengths and limitations

A major strength of this study is the application of the capability approach to the context of physical activity promotion among older men. While the capability approach has previously been successfully examined in the context of well-being [63, 64], its application its application to physical activity provides insights into how individual and socio-structural conditions are associated with perceived opportunities for an active lifestyle. By focusing on capabilities rather than physical activity behavior alone, this study contributes to a broader understanding of the conditions that may enable or constrain active lifestyles among older men.

Furthermore, the study contributes to the empirical examination of the relationships between conversion factors, perceived capabilities, and physical activity behavior. The use of partial least squares structural equation modeling (PLS-SEM) enabled the simultaneous examination of these theoretically derived relationships within the applied framework.

This study has several limitations. First, the sample was recruited from a single rural municipality in Bavaria, Germany, and included exclusively men aged 50 years and older. Therefore, the sociodemographic and contextual characteristics of the sample may differ from those of the broader population of older men in Germany, particularly those living in urban areas. Consequently, the generalizability of the findings to other regions and population groups may be limited. Second, although the sample size of 316 participants allowed for the analyses conducted, larger and more diverse samples would be valuable to further validate the findings and examine potential differences across population subgroups. Future research should include samples from diverse geographical settings and consider incorporating additional variables or complementary qualitative methods to validate the results.

Second, cross-sectional design and reliance on self-reported data constrain the ability to assess causality or agency. Furthermore, all variables were assessed using the same survey instrument, which may introduce potential shared-method bias. Although the use of a theoretically grounded measurement model and the assessment of construct validity provide some safeguards, no specific statistical test for common method bias was conducted. Future research could incorporate multiple data sources or longitudinal designs to further address this issue. Finally, as this study is based on secondary data analysis, future research should apply newly developed instruments tailored for operationalizing the capability approach, such as the updated questionnaire by Boyer et al. [21].

## Conclusions

Through the lens of the capability approach, this study provides valuable insights into the interplay between individual and structural factors in the context of physical activity promotion among older men in rural regions. More favorable individual and structural factors are associated with more perceived capabilities for an active lifestyle, which in turn were associated with physical activity behavior. Future research should further investigate how capabilities relate to physical activity behavior and how supporting equitable capabilities for participation may contribute to a more inclusive and equitable approach to physical activity promotion.

## Data Availability

The dataset supporting the conclusions of this article is available from the corresponding author on reasonable request.

## Appendix

**Table 5:** Content of the questionnaire

Item	Source
Sociodemographic variables	
Age from 50–99 years (coded into two age groups)	[65]
Educational level (coded into three educational groups)	
Living arrangement (coded as living alone vs. living with another person)	[66]
Individual Factors (Self-efficacy with 2 items and 6-point Likert scales, and individual barriers with 13 items and 7-point Likert scales)	
Motivational self-efficacy	[37]
Maintenance self-efficacy	
Individual barriers: No interest/desire	[38]
Individual barriers: Too exhausting	
Individual barriers: Uncertain about physical skills/abilities	
Individual barriers: Laziness	
Individual barriers: No activity in which I feel comfortable	
Individual barriers: Work-related commitments	
Individual barriers: Health-related reasons	
Individual barriers: Difficulty getting started	
Individual barriers: Family obligations/burden	
Individual barriers: Lack of time	
Individual barriers: Fear of inury	
Individual barriers: Lack of time for regular appointments/scheduled activities	
Indiviudal barriers: Feeling ashamed of body appearance	
Structural Factors (11 items with 7-point Likert scales)	
Socio-structural barriers (Opportunities): Few sports facilities/physical activity opportunities	[39]
Socio-structural barriers (Accessibility): No public transport connection	
Socio-structural barriers (Accessibility): Demands of the neighborhood (e.g., steep hills)	
Socio-structural barriers (Accessibility): Design of the neighborhood (e.g., few green spaces)	
Socio-structural barriers: Sports facilities/activities are too expensive	
Socio-structural barriers: Inconvenient schedule	
Socio-structural barriers (Opportunities): Few walking and cycling paths	
Socio-structural barriers (Safety): Lack of safe walking and cycling paths	
Socio-structural barriers: Lack of support or negative attitudes within the neighborhood	
Socio-structural barriers: Lack of support from family	
Socio-structural barriers: Lack of support from the social environment (neighbors, friends)	
Capabilities for an active lifestyle (3 items with a 7-point Likert scale)	
I perceive the opportunity in my life to be physically active in my leisure time as…	[40–42]
I perceive the opportunity in my life to live healthily as…	
I perceive the opportunity in my life to organize my leisure time according to my needs as…	
Physical Activity Behavior – Extent and intensity in leisure & active travel (3 items with each measuring the number of days and duration for at least 10 min)	
Intensive physical activity in leisure time (min. per week)	[43]
Moderate physical activity in leisure time (min. per week)	
Active travel on foot or bicycle (min. per week)

**Table 6:** Test for discriminant validity via the Fornell-Larcker-Criteria

	**IF**	**SF**	**CA**	**PAB**
IF	0.764			
SF	0.387	0.755		
CA	-0.472	-0.403	0.878	
APAB	-0.219	-0.086	0.321	0.704

*IF* Individual factors, *SF* Socio-structural factors, *CA* Capabilities, *APAB* Actual physical activity behavior

**Table 7:** Test for discriminant validity via HTMT-values

	**IF**	**SF**	**CA**	**PAB**
IF				
SF	0.479			
CA	0.523	0.446		
APAB	0.328	0.196	0.465	

*IF* Individual factors, *SF* Socio-structural factors, *CA* Capabilities, *APAB* Actual physical activity behavior
